# Inhibition of Tumor Microenvironment Cytokine Signaling Sensitizes Ovarian Cancer Cells to Antiestrogen Therapy

**DOI:** 10.3390/cancers14194675

**Published:** 2022-09-26

**Authors:** Lijun Tan, Katelyn Tondo-Steele, Caroline Foster, Carrie McIlwain, Danielle E. Bolland, Howard C. Crawford, Andrew Sciallis, Karen McLean

**Affiliations:** 1 Division of Gynecologic Oncology, Department of Obstetrics and Gynecology, University of Michigan, 1500 E. Medical Center Dr, Ann Arbor, MI 48109, USA; 2 Cancer Institute of Chicago, 6700 West 95th Street, Suite 330, Oak Lawn, IL 60453, USA; 3 Science and Math Division, University of Minnesota, 600 East 4th Street, Morris, MN 56267, USA; 4 Henry Ford Pancreatic Cancer Center, Henry Ford Hospital, 2799 W. Grand Blvd, Detroit, MI 48202, USA; 5 Pathology and Laboratory Medicine Institute, Cleveland Clinic Foundation, 9500 Euclid Ave., Cleveland, OH 44195, USA; 6 Department of Gynecologic Oncology and Department of Pharmacology & Therapeutics, Roswell Park Comprehensive Cancer Center, Elm & Carlton Streets, Buffalo, NY 14263, USA

**Keywords:** mesenchymal stem cells, ovarian cancer, leukemia inhibitory factor (LIF), interleukin-6 (IL6), STAT3, JAK2 inhibition, estrogen receptor, antiestrogen therapy (AET)

## Abstract

**Simple Summary:**

Antiestrogen hormonal therapy is a relatively low side effect, orally administered cancer treatment option, yet response rates have been limited in epithelial ovarian cancer despite estrogen receptor expression in many tumors. This suggests that other pathways impact estrogen response. Cytokine signaling from the tumor microenvironment promotes ovarian cancer growth, and crosstalk between cytokine signaling and estrogen signaling has been reported in other tumor types. We therefore aimed to investigate whether cytokine signaling impacts estrogen signaling in high-grade serous ovarian cancer. We demonstrated crosstalk between these two pathways in patient-derived samples, in vitro and in animal studies. We found that inhibiting interleukin-6/leukemia inhibitory factor (IL6/LIF) cytokine signaling activates estrogen signaling and blocking both pathways was synergistic in inhibiting tumor cell growth. These results suggest a potential role for combination therapy for epithelial ovarian cancer patients.

**Abstract:**

Antiestrogen therapy (AET) is an alternative to cytotoxic chemotherapy for recurrent ovarian cancer, yet the often short duration of response suggests mechanisms of resistance. We previously demonstrated that tumor microenvironment interleukin-6/leukemia inhibitory factor (IL6/LIF) cytokines induce tumor cell JAK-STAT signaling to promote cancer growth. Crosstalk between estrogen signaling and cytokine signaling has been reported. Therefore, we sought to characterize the impact of IL6/LIF signaling on estrogen signaling in epithelial ovarian cancer and investigate the efficacy of combination therapy. We first assessed patient tumors for cytokine expression and compared it with response to AET to determine clinical relevance. In vitro, we determined the effect of IL6/LIF on estrogen receptor expression and signaling. Cell viability assays were used to determine the efficacy and potential synergy of cytokine blockade and AET. We then extended studies to animal models, incorporating patient-derived stromal cells. Our results demonstrated shorter progression-free interval on AET in patients with stromal IL6/LIF expression. In vitro, IL6/LIF increased tumor cell estrogen receptor expression and signaling, and combination cytokine blockade and AET resulted in synergistic inhibition of tumor cell growth. The anticancer effect was verified in a mouse model. In conclusion, due to crosstalk between IL6/LIF cytokine signaling and estrogen signaling, dual blockade is a potential new treatment approach for ovarian cancer.

## 1. Introduction

Ovarian cancer is the fifth highest cause of cancer deaths among women in the United States, with a 5-year survival rate of less than 50%. Cancers of ovarian, fallopian tube and primary peritoneal origins are frequently collectively termed “ovarian cancer”, with high-grade serous carcinoma (HGSC) being the most common and most lethal epithelial subtype. Most HGSC patients initially respond to cytotoxic chemotherapy, but later develop recurrent disease that is resistant to standard treatments. Platinum-resistant ovarian cancer is generally treated with single agent cytotoxic chemotherapy with palliative intent [1], with objective response rates ranging from 10–30% and a median progression-free interval (PFI) of 3–5 months [2,3]. Given this limited efficacy and the potential for significant side effects with cytotoxic chemotherapy agents, it is imperative to develop new treatment approaches that improve efficacy and/or prioritize quality of life factors.

One potential alternative to cytotoxic chemotherapy for the treatment of HGSC is a hormonal approach utilizing antiestrogen therapy (AET). We previously reported a cohort of heavily pre-treated ovarian cancer patients with a median PFI of 4.0 months on AET, including responses in some patients with ER-negative tumors [4]. These data are in alignment with the finding that approximately 80% of epithelial ovarian cancers express estrogen receptor-alpha (ERα) [5], yet a 20% response rate to AET is noted in clinical studies [6]. Together, these findings suggest modifiers of response that can potentially be targeted to improve hormonal therapy response rates in HGSC.

Ovarian cancer-associated mesenchymal stem cells (CA-MSC) in the tumor microenvironment promote tumorigenesis through the secretion of factors including interleukin-6 (IL6) and the related cytokine leukemia inhibitory factor (LIF) [7,8]. IL6 cytokine expression has been shown to be inversely correlated with AET response in ovarian cancer cell lines [9,10]. Stromal IL6 signaling has been shown to alter hormone metabolism in ER-negative breast cancers [11] and crosstalk between estrogen and IL6 signaling in the tumor microenvironment has been reported in prostate cancer cell lines [12]. LIF signaling has also been implicated in tumorigenesis, and we previously demonstrated that LIF functions in parallel with IL6, thus requiring dual cytokine blockade for optimal antitumor effects [8]. Furthermore, IL6 has been shown to regulate and enrich ovarian cancer stemness following platinum-based therapy [13].

Based on these clinical and preclinical data, we hypothesized that there is crosstalk between cytokine signaling and estrogen signaling pathways in HGSC; thus, inhibition of cytokine signaling including that from the tumor microenvironment may sensitize ovarian cancers to AET. Herein, we report on the ability of IL6/LIF cytokine signaling to increase ER signaling and the use of dual cytokine and estrogen blockade to decrease HGSC cell growth in vitro synergistically as well as in vivo in animal models. These findings suggest a novel combination therapy for the treatment of ovarian cancer.

## 2. Materials and Methods

### 2.1. Cell Lines

Ovarian cancer cell lines OVSAHO, SKOV3, COV362, CaOV3 and breast cancer cell lines T47D and MCF7 were obtained from the American Type Culture Collection (ATCC). OVCAR3 and OVCAR4 were obtained from the National Cancer Institute. SNU119 was purchased from AcceGen Biotechnology (Fairfield, NJ, USA). Cell line identity was validated by STR profile report using the ATCC Cell Line Authentication service. Cell lines were maintained in standard culture conditions. Mycoplasma testing was performed (PlasmoTest Kit, Invivogen, San Diego, CA, USA) and confirmed to be negative at least every two months to maintain healthy cell cultures.

### 2.2. Patient-Derived Samples

Primary, patient-derived specimens from women undergoing surgery at our institution were obtained through institutional review board-approved protocols (HUM00125624, HUM00148299). Cancer-associated mesenchymal stem cells (CA-MSC) were isolated and maintained as previously described [7,8]. For conditioned media experiments, cells were cultured in serum-free media for 24 h prior to collection of media. As controls, non-cancer human adipose-derived mesenchymal stem cells were purchased from ATCC (Cat. No. PCS-500-011) or Invitrogen (Cat. No. R7788115) (Manassas, VA, USA).

### 2.3. Immunohistochemical Analysis of Primary Patient Tumors

Under an institutional review board-approved protocol (HUM00072411), patients with a history of ovarian cancer and AET were identified. Clinical outcomes were obtained through data extraction from the electronic medical record and correlated with ERα expression as previously reported [4]. For that study, ERα immunohistochemistry was performed with the antibody clone 1D5 (BIOCARE, Pacheco, CA, USA, Cat. No. ACA054A) and an Allred score of 0–8 applied, with a score ≥ 3 indicating ERα positivity. For the present study, formalin-fixed, paraffin-embedded tumor specimens were assessed by immunohistochemistry for IL6 expression (primary antibody from Abcam, Cambridge, UK, Cat. No. ab9324) and LIF expression (primary antibody from Abcam, Cat. No. ab135629). Staining was scored by a board-certified pathologist, and positivity was determined based on the presence of granular or diffuse staining within the cytoplasm and nucleus of the cells of interest. IHC for both IL6 and LIF was then analyzed via a combination of the aggregate percentage of stained cells and the intensity of cell staining (0 = negative; 1+ = mild; 2+ = moderate; 3+ = strong). Presence or absence of stromal staining was utilized for survival analyses.

### 2.4. Cell Viability Assays

Media was changed to 2% charcoal-stripped fetal bovine serum (FBS) at 24 h and then cells were plated in 96 well plates. After 24 h, fresh media with 2% charcoal-stripped FBS was placed without or with the following drugs: tamoxifen (Sigma, St. Louis, MO, USA, T5648), letrozole (Sigma, L6545) or ruxolitinib (Selleckchem, Radnor, PA, USA, S1378). Cell viability was determined with the MTT Cell Viability Assay Kit (Biotium, Fremont, CA, USA, Cat. No.30006).

### 2.5. Reporter Assays

The ERE Cignal Reporter Assay Kit (QIAGEN, Hilden, Germany, Cat. No. CCS-005L) was used per the manufacturer’s instructions, with plasmid complexes transfected into the cells with Lipofectamine 2000 in OptiMEM media 10% charcoal-stripped FBS. After 6 h, media was changed to standard media with 2% charcoal-stripped FBS without antibiotics and without or with the indicated treatment of estradiol, IL6, LIF, IL6 + LIF, or mock treatment. After 24 h of treatment, luciferase signal intensities were quantified per the manufacturer’s protocol (Dual-Glo Luciferase Assay System, Promega Cat. No. E2920) and reporter assay activation calculated compared to controls.

### 2.6. Immunoblotting

To assess basal ER expression levels, cells were cultured in standard conditions and protein lysates harvested in RIPA lysis buffer (Sigma, R0278). To determine the impact of cytokine signaling on ER expression levels, tumor cells were treated with either CA-MSC condition media, or serum-free media containing 50 ng/mL recombinant IL6 (4-8069-80, eBioscience) and/or 50 ng/mL recombinant LIF treatment (14-8460-80, eBioscience) for 24 h. RIPA lysates were prepared and subjected to immunoblotting with antibodies to ERα (Abcam, Cat. No. ab116716), followed by secondary antibody (Cell Signaling, Danvers, MA, USA, Cat. No. 7074S). The membrane was developed with Pierce ECL solution (Thermo Scientific, Waltham, MA, USA, 23106). GAPDH immunoblotting was utilized as a loading control.

### 2.7. Colony Formation Assay

Ovarian cancer cells were seeded in 24-well plates (200 cells/well, in triplicates) and cells were treated with vehicle control, ruxolitinib, letrozole and ruxolitinib/letrozole in combination at the indicated concentrations for two weeks to allow for colony formation. Medium was changed every third day. Visible colonies were fixed with 4% paraformaldehyde and stained with 0.005% crystal violet at RT for 5 min following distilled water washing. The number of colonies per well was counted.

### 2.8. Animal Studies

All studies were performed under institutional animal care and use committee (IACUC)-approved protocols and regulations. Female NSG mice were obtained from Jackson Laboratories (strain 005557). An initial pilot experiment was performed to characterize tumor growth rates and dosing schedules, followed by a second experiment to quantify treatment effects. At approximately eight weeks of age, the mice underwent bilateral ovariectomy. After two weeks of healing, mice were injected subcutaneously with 0.5 × 10^6^ OVCAR3 human epithelial ovarian cancer cells and 0.5 × 10^6^ patient-derived CA-MSC suspended in Matrigel. Tumors reached approximately 200 mm^3^ after ten days; animals were then randomized to treatment groups by even distribution of starting weights and tumor size (*n* = 5 per group, sample size selected empirically). Cohorts were treated with (1) control chow (Incyte Corp., Palo Alto, CA, USA), (2) ruxolitinib chow (2 g/kg chow, Incyte Corp.), (3) control chow plus letrozole or (4) ruxolitinib chow plus letrozole. Letrozole was dissolved in 2% DMSO w/98% PBS (L6545 Sigma) and 20 ug daily administered intraperitoneally. Animals were sacrificed at either > 10% weight loss or significant illness, as discussed with the animal care facility.

### 2.9. Statistical Analysis

All experiments were performed in duplicate or triplicate, and all data are expressed as the mean ± standard deviation. Statistical analysis was performed using GraphPad Prism version 8.0 for Windows (GraphPad Software, San Diego, CA, USA). For single comparisons, an unpaired, two-tailed t test was used. For multiple comparisons, one-way analysis of variance (ANOVA) with Tukey’s or Bonferroni post hoc test was performed. For survival curve comparisons, statistical analysis was performed with the log-rank Mantel-Cox method. Results were considered statistically significant with a *p* value of ≤0.05. For all figures, * *p* < 0.05, ** *p* < 0.01, *** *p* < 0.001, and **** *p* < 0.0001.

## 3. Results

### 3.1. Stromal IL6/LIF Cytokine Expression Is Correlated with a Shorter Progression-Free Interval on Antiestrogen Therapy

**Figure 1:**
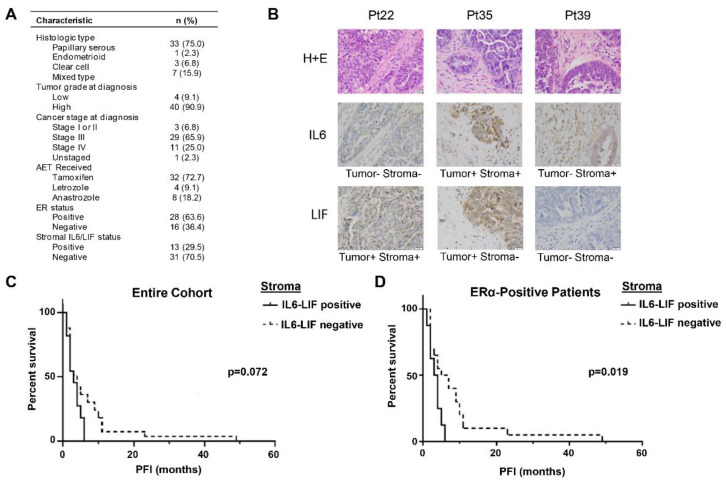
The role of ERα and IL6/LIF expression in response to AET. For a cohort of patients treated with AET, tumors were stained for ERα expression by IHC and staining scored using the Allred method. (**A**) Characteristics of patients treated with AET. (**B**) Representative histologic findings, including positive and negative staining. (**C**) Progression-free interval on AET in IL6/LIF stroma positive versus negative tumors for entire cohort and (**D**) Progression-free interval on AET in IL6/LIF stroma positive versus negative tumors for patients with ERα positive tumors. Statistical analysis performed with the log-rank Mantel-Cox method; *p* values shown.

To define a clinical relevance for the relationship between IL6/LIF expression and response to AET, we examined the expression of ERα, IL6 and LIF in tumor specimens derived from a cohort of patients with ovarian, Fallopian tube or primary peritoneal cancer treated with AET (Figure 1A) [4] and assessed the correlations between expression patterns and PFI. The majority of the patients in the cohort had advanced stage, high-grade serous carcinoma. Approximately 73% received tamoxifen as their AET, 9% received the aromatase inhibitor letrozole and 18% received the aromatase inhibitor anastrozole.

Tumor ERα expression was previously scored using the validated Allred method [4,14]. Immunohistochemistry was performed to assess IL6 and LIF expression in both the tumor cells and surrounding stroma. Stained tumor slides were scored by a board-certified pathologist; representative positive and negative staining are shown (Figure 1B). Given the redundancy in IL6/LIF signaling through the GP130 coreceptor and downstream JAK/STAT signaling cascade, tumors were scored as positive if they expressed either cytokine in the tumor microenvironment.

In the entire cohort of patients (*n* = 44) treated with AET, we found that patients with IL6/LIF stroma-positive tumors (*n* = 13) trended toward a shorter mean PFI (4.3 months) than patients with IL6/LIF-negative tumors (*n* = 31, mean PFI = 6.4 months), *p* = 0.072 (Figure 1C). We then examined the subset of patients with ERα-positive tumors (*n* = 28). Amongst this subset of patients, those with tumors that expressed IL6/LIF in the stroma (*n* = 9) had a statistically significant shorter mean PFI (4.1 months) compared to patients with tumors that lacked IL6/LIF expression in the stroma (*n* = 19, mean PFI = 8.6 months), *p* = 0.019 (Figure 1D). Collectively, these data suggest that expression of IL6/LIF in the tumor microenvironment is negatively correlated with response to AET.

### 3.2. IL6 and LIF Signaling Increase Tumor Cell ERα Expression

**Figure 2:**
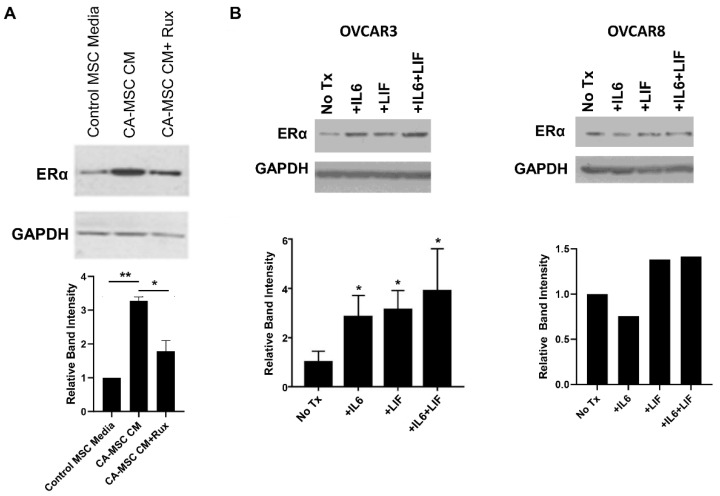
IL6 and LIF signaling from CA-MSC increases ERα expression in the HGSC cell lines. (**A**) OVCAR3 cells were exposed to control media or conditioned media from CA-MSC (CA-MSC CM) and either mock treated or treated with the JAK1/2 inhibitor ruxolitinib (+Rux) and ERα protein immunoblotting was performed. GAPDH was used as a loading control. Two independent experiments were performed; band intensities were quantified with Image J and normalized to GAPDH. (**B**) OVCAR3 and OVCAR8 cells were treated as indicated (IL6 and LIF at 50 ng/mL) and immunoblotting performed for ERα and GAPDH. Three independent experiments were performed for OVCAR3 and one experiment for OVCAR8, and band intensities quantified. Results were compared to no treatment with two-tailed Student *t*-test. * *p* < 0.05, ** *p* < 0.01. The uncropped blots are shown in Figure S1.

To test our hypothesis that cytokine signaling from the ovarian cancer tumor microenvironment impacts tumor cell estrogen pathways, we assessed the impact of CA-MSC conditioned media on ERα expression by immunoblotting. High-grade serous carcinoma OVCAR3 cells were grown in one of three mesenchymal media conditions: (1) control MSC media, (2) media that had been collected following culture with CA-MSC termed conditioned media (CM), or (3) CA-MSC CM treated with the JAK inhibitor ruxolitinib (Figure 2A). ERα expression was noted to increase with CA-MSC conditioned media when compared to control MSC media, suggesting that CA-MSC secrete factors that upregulate tumor cell ERα expression. Furthermore, treatment with the JAK inhibitor ruxolitinib resulted in blockade of CA-MSC CM induction of ERα upregulation, implicating the JAK/STAT pathway as the critical signaling pathway (Figure 2A).

To directly assess the impact of IL6/LIF signaling on ERα expression, OVCAR3 and OVCAR8 cells were treated with recombinant IL6 and LIF either alone or in combination, followed by immunoblotting to determine ERα expression. In OVCAR3 cells, all three cytokine treatments resulted in an increase in ERα protein, with combination therapy demonstrating the highest expression of ERα (Figure 2B). Three independent experiments were performed; quantification of band intensities demonstrated a statistically significant increase in relative band intensity with either single or combination cytokine therapy compared to no treatment (*p* < 0.05 for all comparisons). Findings in OVCAR8 cells also suggest induction of ERα expression following combination cytokine treatment.

### 3.3. IL6/LIF Induces Increased Estrogen Reporter Assay Activation in ERα-High Cell Lines

**Figure 3:**
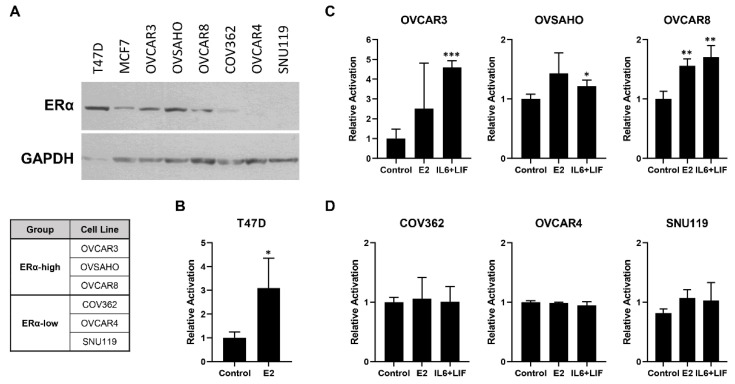
ERα expression screening by immunoblotting and ERE activities by luciferase reporter assay in HGSC. (**A**) Immunoblotting for was performed on a panel of HGSC cell lines as well as the ERα-positive breast cancer cell lines T47D and MCF7. HGSC lines were classified into ERα-high and ERα-low categories as shown. The ERE Cignal Reporter Assay Kit (QIAGEN) was used per the manufacturer’s instructions, with ERE plasmid complexes transfected into the cells with Lipofectamine 2000 in OptiMEM media 10% charcoal-stripped FBS. Cells were treated with estradiol (10 nM), IL6 (50 ng/mL), LIF (50 ng/mL), IL6 + LIF, or mock treatment. After 24 h of treatment, luciferase signal intensities were quantified and reporter assay activation calculated compared to controls. (**B**) T47D breast cancer cells were served as positive control showing higher luciferase reporter activation following estradiol (E) treatment. (**C**) IL6 + LIF increased luciferase reporter activation in ERα-high HGSC cell lines OVCAR3, OVSHAO and OVCAR8 but not in (**D**) ERα-low HGSC cell lines COV362, OVCAR4 and SNU119. * *p* < 0.05, ** *p* < 0.01, *** *p* < 0.001. The uncropped blots are shown in Figure S2.

To expand our model systems for interrogation of ER signaling in high-grade serous cancer, we performed immunoblotting to assess ERα expression in a panel of established high-grade serous cell lines, with ERα-positive breast cancer cell lines serving as positive controls (Figure 3A). Based on receptor expression levels, we classified tumors into either high or low levels of ERα. OCVAR3, OVSAHO and OVCAR8 were classified as ERα-high and COV362, OVCAR 4 and SNU119 were classified as ERα-low (Figure 3A).

We then assessed both the potential activation of estrogen signaling in response to estradiol (E2) or cytokine treatment utilizing a luciferase reporter construct driven by estrogen response elements (ERE). The ERα-positive breast cancer cell line T47D served as a positive control for estrogen signaling following E2 treatment (Figure 3B). Cell lines were transfected with the ERE reporter construct and either mock treated or treated with either E2 or IL6/LIF. Combinatorial therapy was utilized given our prior studies demonstrating redundancy of the pathways in impacting HGSC cell signaling [8]. ERE reporter activation following E2 treatment mirrored ERα expression, with activation inOVCAR3, OVSAHO and OVCAR8 cell lines (Figure 3C). The ERα-low high-grade serous lines COV362, OVCAR4 and SNU119 did not demonstrate ERE reporter activation following E2 therapy (Figure 3D). In the ERα-high HGSC cell lines (Figure 3C), there was a statistically significant increase in luciferase reporter activation when treated with IL6 + LIF (*p* < 0.001 for OVCAR3, *p* = 0.046 for OVSAHO, and *p* < 0.007 for OVCAR8). In comparison, the ERα-low cell lines COV362, OVCAR4 and SNU119 did not show an increase in luciferase ERE reporter activation following IL6 + LIF therapy (Figure 3D). These findings support IL6/LIF activation of ER signaling in ERα-positive high-grade serous tumors.

### 3.4. Letrozole and Ruxolitinib Demonstrate Synergy in Ovarian Cancer Cell Lines

**Figure 4:**
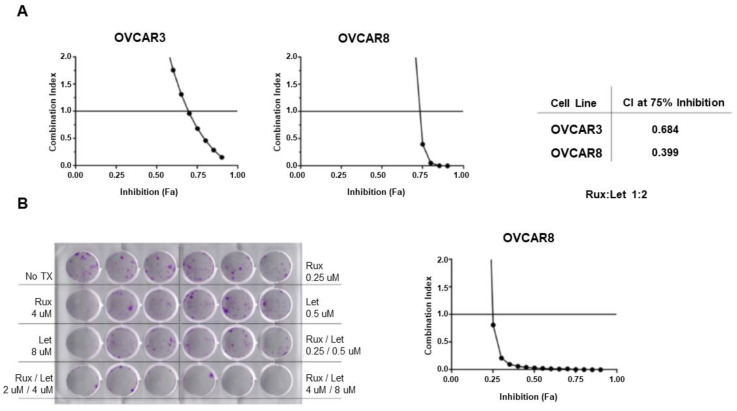
Letrozole and ruxolitinib demonstrate synergy in ovarian cancer cell lines. ERα-high ovarian cancer cell lines OVCAR3 and OVCAR8 were treated with a range of doses of ruxolitinib (Rux) and letrozole (Let) at ratio of 1:2 and cell viability quantified by MTT assay. The combination index (CI) was determined using Compusyn applying the Chou-Talalay method. (**A**) The CI relative to the fraction affected (Fa) is shown for OVCAR3 and OVCAR8. CI at 75% inhibition for each cell line is shown, with values < 1 indicating synergy. (**B**) Colony formation assay for OVCAR8 and graphical representation demonstrating synergy in the colony formation assay.

After establishing the ability of cytokines IL6/LIF to activate ERα expression and estrogen signaling, we sought to examine the effect of combination therapy blocking both pathways on cell viability. We investigated the impact of cytokine signaling inhibition using the JAK2 inhibitor in combination with the aromatase inhibitor letrozole. Letrozole was selected as the antiestrogen for further studies based on its preferred use in clinical practice at this time and its oral bioavailability for translational studies. ERα-high ovarian cancer cell lines OVCAR3 and OVCAR8 were treated with a range of doses of ruxolitinib and letrozole at a 1:2 ratio and cell viability was quantified. Combination index was calculated using the Chou-Talalay method [15], with a combination index of <1 indicating synergy. In both OVCAR3 and OVCAR8, the combination of ruxolitinib and letrozole resulted in synergistic cell killing (Figure 4A).

To validate these results, colony formation assays were performed with ruxolitinib alone, letrozole alone, and combination treatment of ruxolitinib and letrozole at different concentrations, again employing the 1:2 ratio. The combination of ruxolitinib and letrozole showed a visible inhibition in colony number compared to control and single drug wells (Figure 4B). Combination index was determined based on the colony formation assays, again demonstrating synergy (Figure 4B).

### 3.5. Combination Therapy with AET and Cytokine Blockade Improves Treatment Response In Vivo

**Figure 5:**
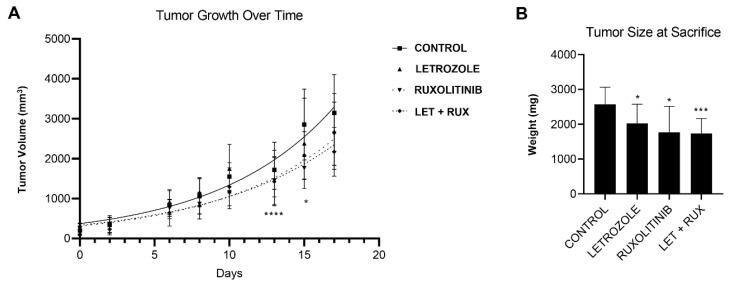
Combination therapy with AET and cytokine blockade improves treatment response in vivo. Ovariectomized mice were injected subcutaneously with OVCAR3 tumor cells and patient-derived CA-MSC. Following tumor establishment, treatment was commenced with groups (*n* = 5 animals each) of control chow, ruxolitinib chow, letrozole intraperitoneally, or ruxolitinib chow plus letrozole intraperitoneally. (**A**) Tumors were monitored, and tumor volume calculated to generate growth curves. Day 0 represents start of treatment. (**B**) At the time of sacrifice, final tumor wights were determined. * *p* < 0.05, *** *p* < 0.001, and **** *p* < 0.0001.

We next sought to extend our findings in vivo by assessing the impact of combination therapy, consisting of cytokine blockade and AET, on tumor growth in a mouse model system. Following an initial pilot experiment to establish methodology and time course, female mice were ovariectomized and injected with OVCAR3 cells plus patient-derived CA-MSC. Animals were then divided into four cohorts (*n* = 5 animals each, with bilateral tumors for 10 evaluable tumors per cohort) and treated as follows: (1) control chow, (2) ruxolitinib chow, (3) control chow plus letrozole or (4) ruxolitinib chow plus letrozole. Tumor growth curves and final tumor weights were assessed; both were consistent with decreased tumor burden in both single agent groups and combination therapy groups compared to controls (Figure 5A,B). At time of animal sacrifice, all treatments showed a statistically significant difference in final tumor weight when compared to the control group. Combination therapy with letrozole plus ruxolitinib did not show statistically significant decreases in tumor size as compared to the single agent group. However, the letrozole plus letrozole group had the lowest average tumor weight (1732 mg), which was statistically significant compared to the control group (2574 mg, *p* < 0.001) (Figure 5B).

## 4. Discussion

Recurrent ovarian cancer is a clinical challenge requiring new treatment approaches that consider the optimization of patient quality of life. Despite most epithelial ovarian cancers expressing ER, clinical response rates to AET have been disappointing. Based on preliminary data and published reports supporting a role for cytokine and estrogen signaling crosstalk, we sought to test the hypothesis that there is an interplay between IL6/LIF cytokine signaling in the tumor microenvironment and estrogen signaling.

We analyzed primary patient tumors and found that patients with tumors in which the stroma expresses IL6/LIF cytokines had shorter PFI on AET, with a statistically significant decrease in PFI specifically in ER-positive tumors. We then mechanistically linked this stromal IL6/LIF expression with tumor ER signaling by demonstrating that IL6/LIF signaling both increases tumor cell ERα expression and induces ER reporter gene activation. Following this demonstration of an interplay between cytokine and estrogen signaling, we demonstrated that blockade of both pathways with the JAK inhibitor ruxolitinib in combination with the aromatase inhibitor letrozole resulted in synergistic cell killing in vitro. Letrozole was selected due to its common clinical use currently and oral bioavailability for patients. Furthermore, we postulate that letrozole blocks estrogen synthesis to decrease estrogen signaling and break the feed-forward loop between estrogen and IL6/LIF cytokine signaling in the ovarian cancer tumor microenvironment. Finally, we performed mouse model studies revealing that combined therapy with AET and cytokine blockade improves treatment response in vivo. These in vivo studies did not show statistically significant decreases with combination therapy as compared to single agent treatments; potential explanations include the rapid tumor growth of the cell line utilized and/or the drug treatment doses utilized.

A primary strength of this study is the inclusion of CA-MSC to investigate the impact of the tumor microenvironment on response to AET in ovarian cancer. Our findings are consistent with other work demonstrating that tumor-stroma interactions lead to production of growth factors capable of activating ERs through a ligand-independent pathway resulting in tumor proliferation [16]. We also incorporated patient-derived CA-MSC in our animal models to recapitulate the tumor stroma. An additional strength of our work is the analysis of primary, patient-derived specimens that demonstrate a link between stromal cytokine expression and response to AET.

This study also has several limitations that will serve to drive future directions. Larger patient sample numbers will allow more robust conclusions; we are currently generating a tissue microarray of primary patient tumors to allow this analysis. Furthermore, antiestrogens with different mechanisms of action than the aromatase inhibitors studied herein may also have unique effects on cytokine-estrogen crosstalk, and studies on selective estrogen degraders (SERDs) would be beneficial. Continued in vivo preclinical studies are also necessary to optimize both the optimal antiestrogen agent and dosing to achieve synergy as compared to single agent treatment in animal models.

Additionally, our current study does not address the role of the immune component of the tumor microenvironment in cytokine signaling and response to therapy. Prior work has demonstrated that LIF and IL6 cytokines in ovarian cancer ascites promote the differentiation of monocytes into immunosuppressive tumor-associated macrophages [17,18]. These findings suggest that cytokine blockade such as that proposed herein would improve treatment responses. Additionally, other studies have revealed that ER signaling functions to suppress immune function in tumors [19,20]. Thus, combination therapeutic approaches incorporating AET would, if anything, be postulated to enhance antitumor immune responses. Collectively, these links warrant further studies in immunocompetent mouse model systems or humanized mouse models that would allow integration of both patient-derived MSC and immune cells.

## 5. Conclusions

In conclusion, the findings reported herein demonstrate a link between IL6/LIF cytokine signaling in the ovarian cancer tumor microenvironment and tumor cell estrogen signaling. Dual blockade of both cytokine signaling and estrogen signaling has the potential to block this crosstalk, improving antitumor responses. Additional preclinical studies will allow refinement of this treatment approach and subsequent translation to clinical trials, with the goal of improving ovarian cancer patient outcomes.

## Data Availability

The data presented in this study are available on request from the corresponding author.

## Supplementary Materials

The following supporting information can be downloaded at: https://www.mdpi.com/article/10.3390/cancers14194675/s1, Figure S1, uncropped blots from Figure 2; Figure S2, uncropped blots from Figure 3.
