# Supplementary material for: Inhibition of Tumor Microenvironment Cytokine Signaling Sensitizes Ovarian Cancer Cells to Antiestrogen Therapy
Source: Cancers (Basel). 2022 Sep 26;14(19):4675. doi: 10.3390/cancers14194675 (PMC9564160; doi:10.3390/cancers14194675)
Supplement: Supplementary file 1 [file cancers-14-04675-s001.zip › cancers-1848681-supplementary.pdf]

Supplemental Figure S1

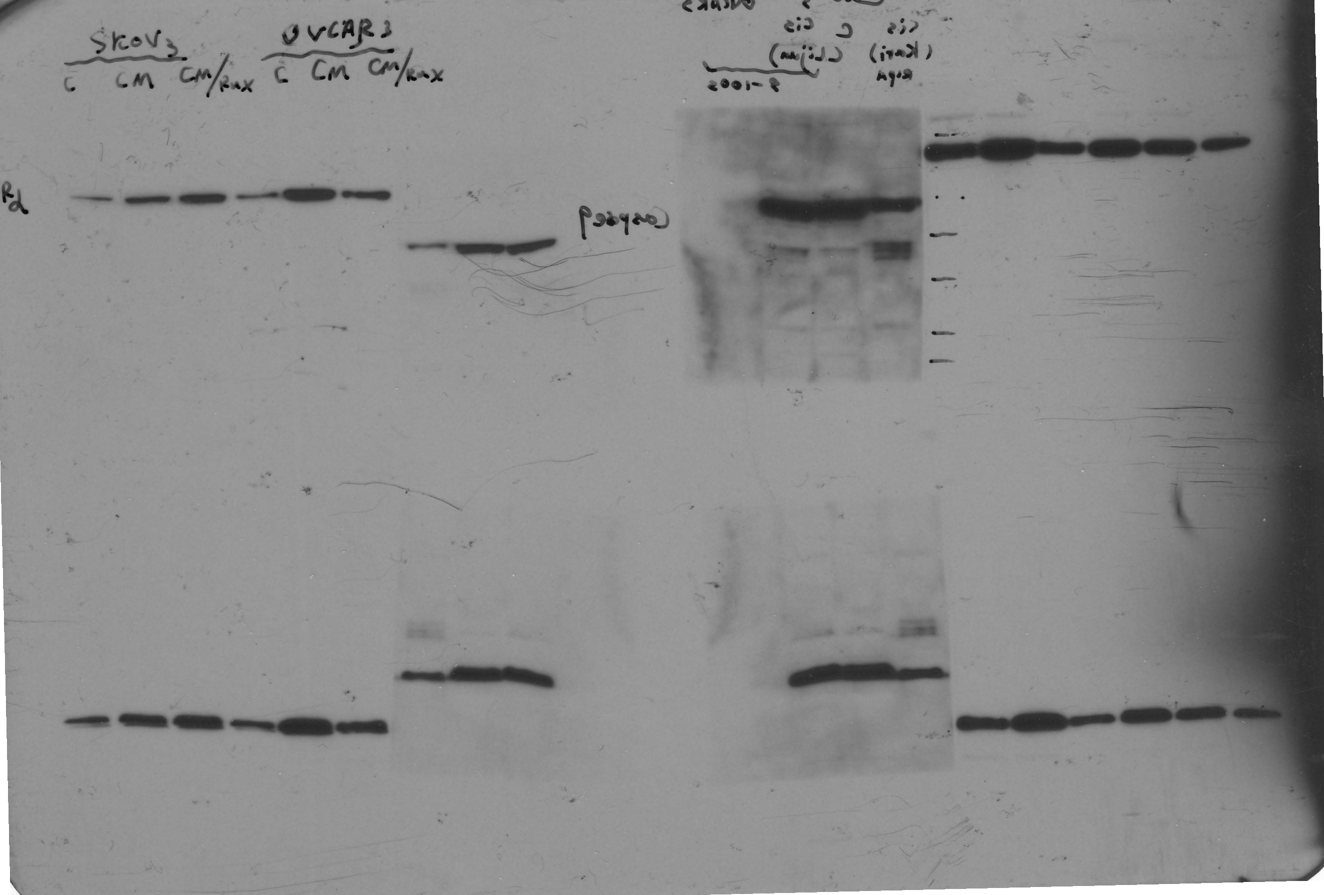

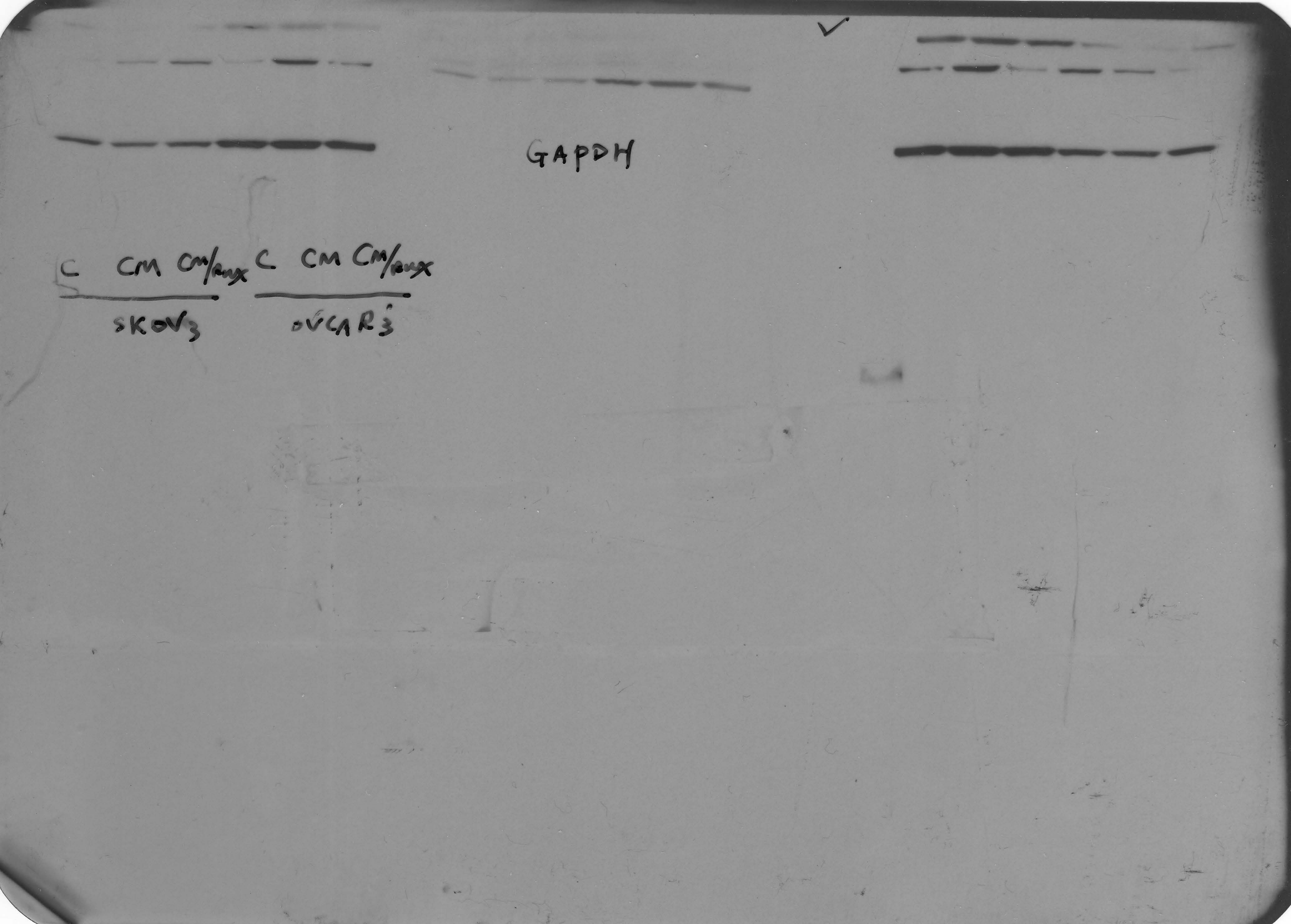
GAPDH
C CM
C CM
SKOV3
OVCAR3

OVCAR8 OVCAR3

6/18/15

OVCAR8				OVCAR3			
No Tx	IL6	LIF	IL6+LIF	No Tx	IL6	LIF	IL6+LIF

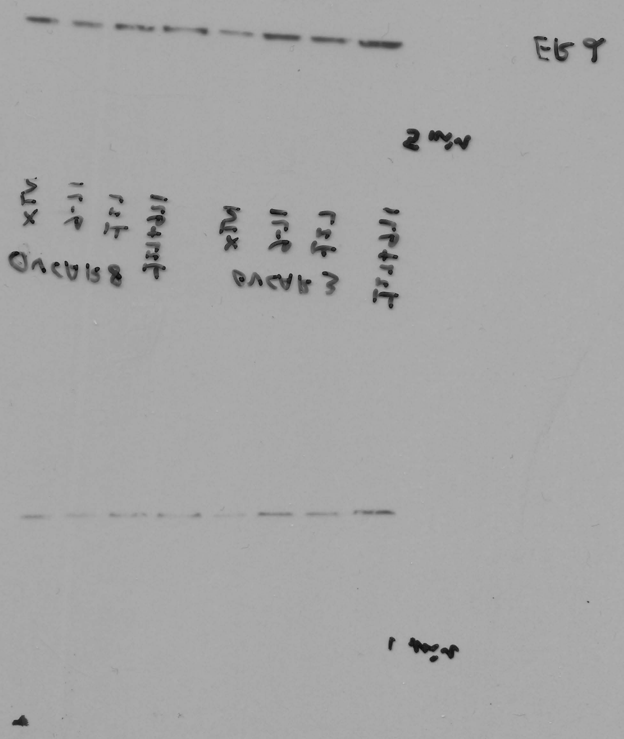

Membrane flipped bottom to top during multiple exposures with single film

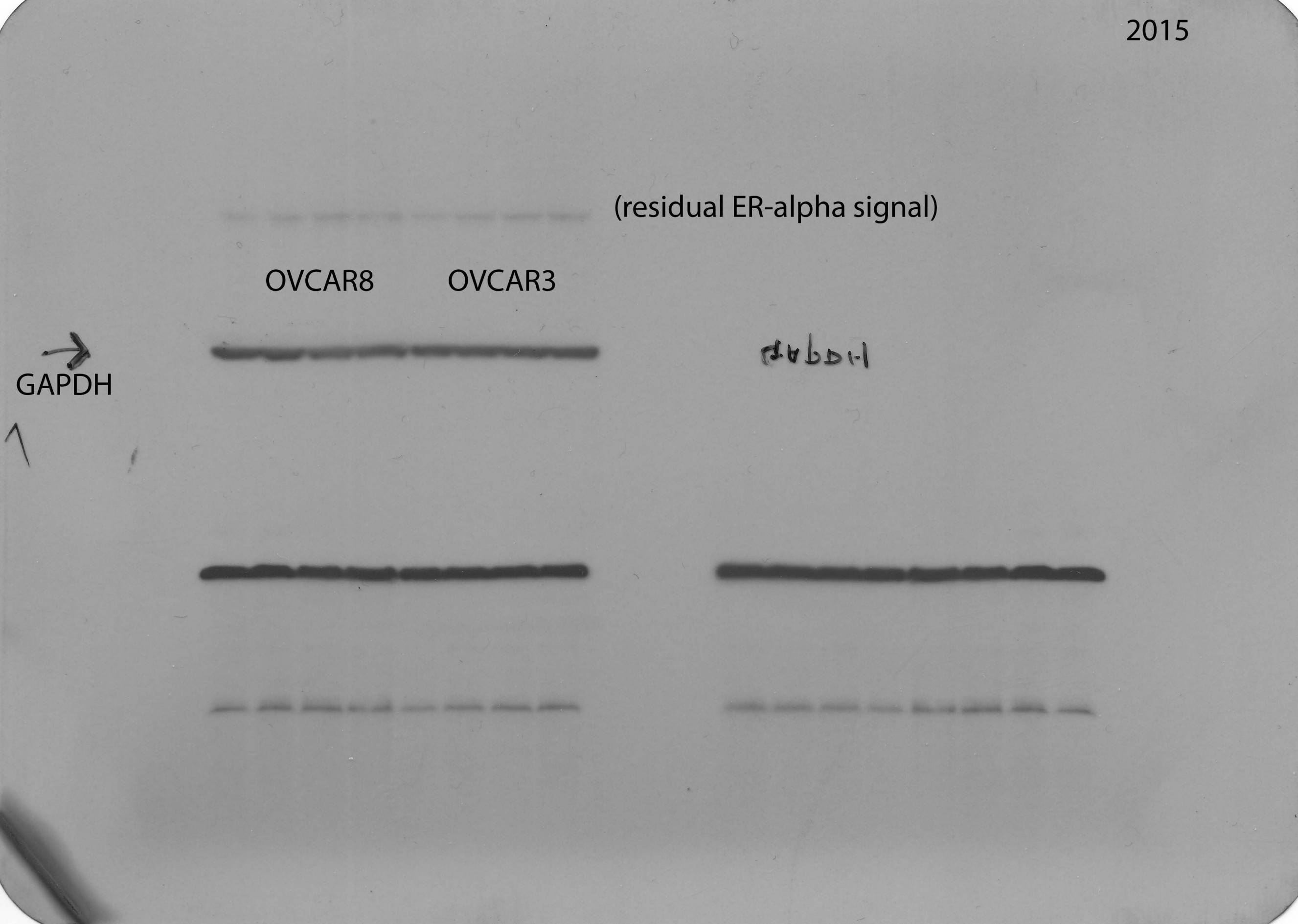
2015
(residual ER-alpha signal)
OVCAR8
OVCAR3
GAPDH

Supplemental Figure S2

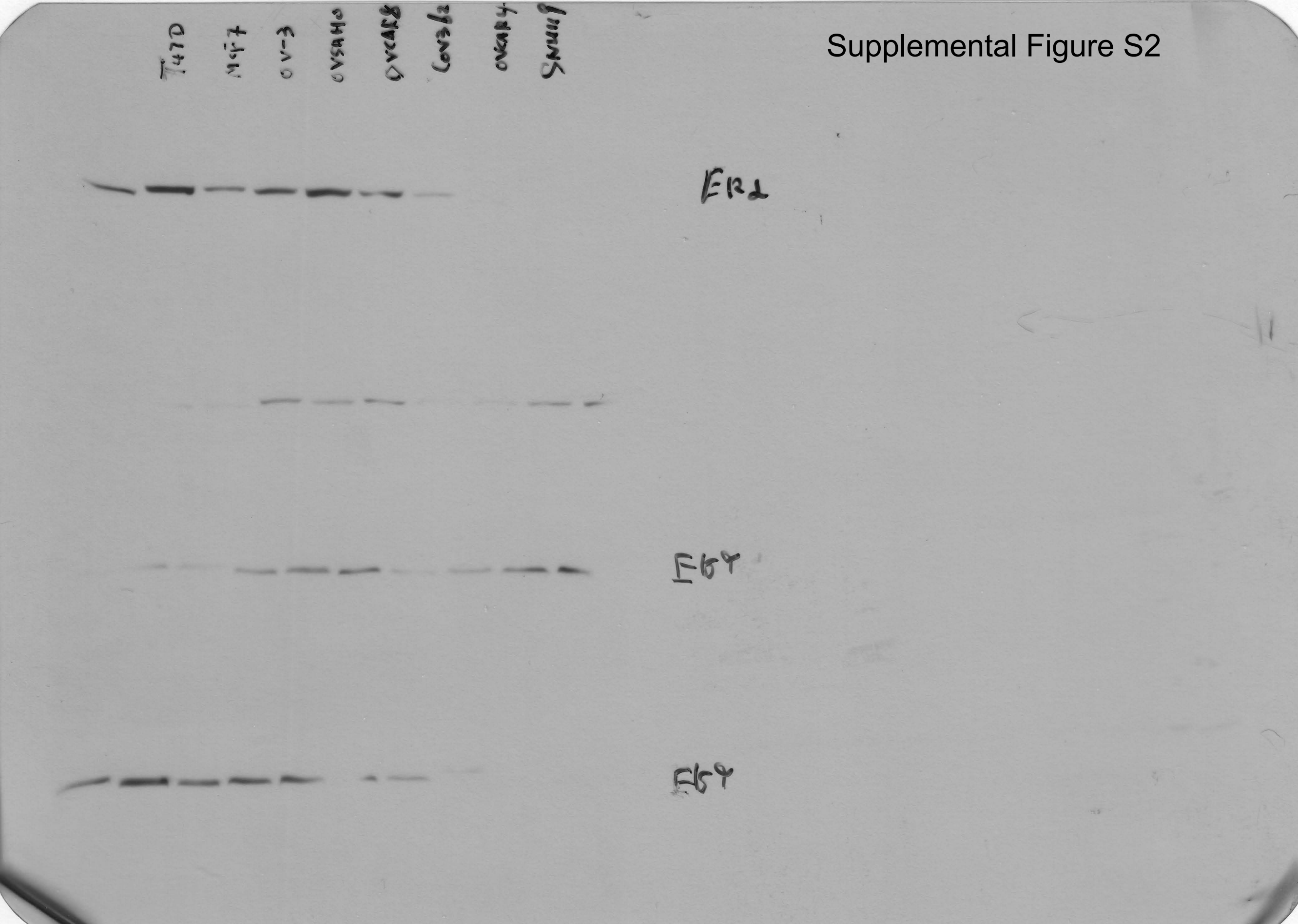

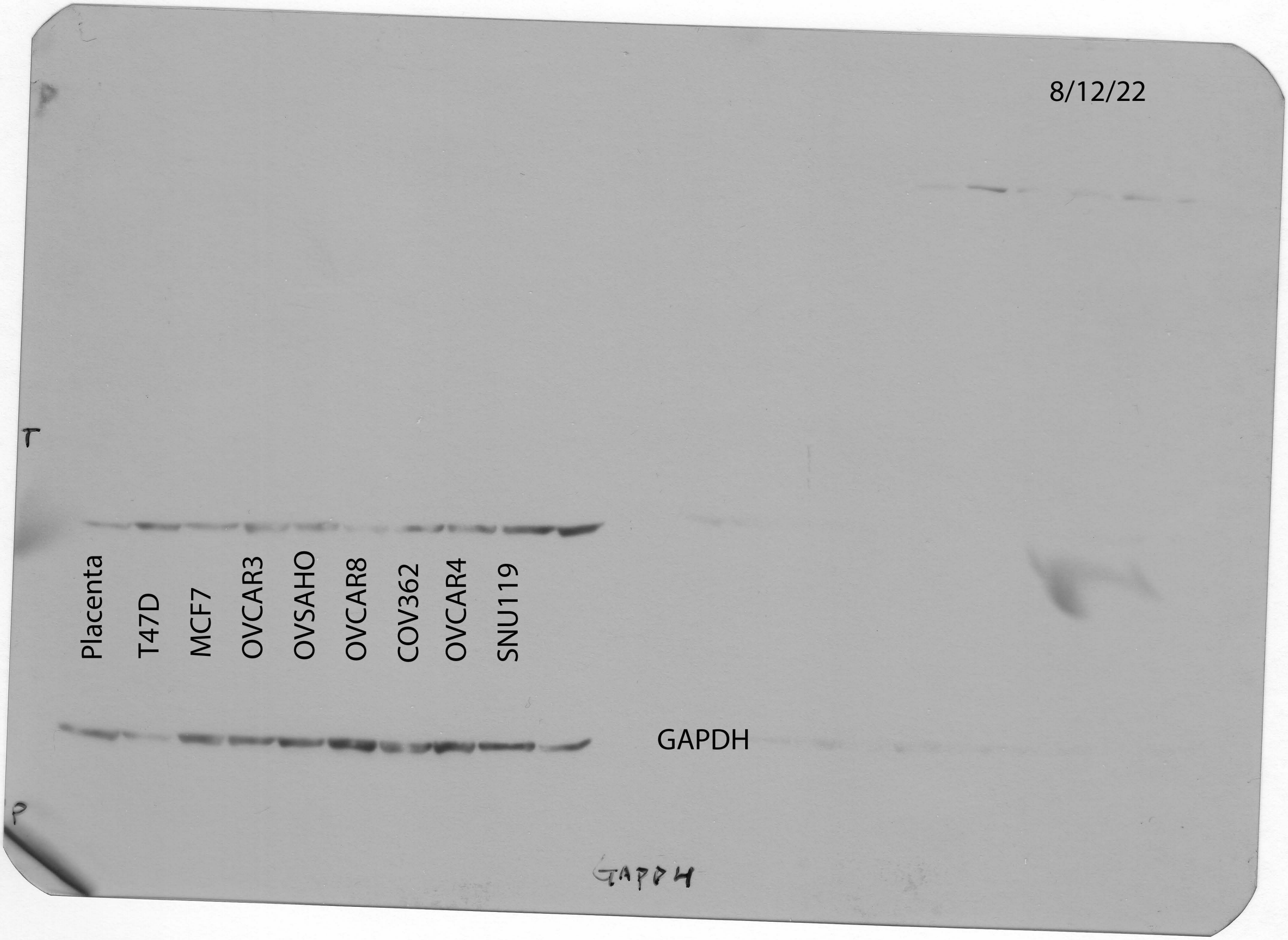
8/12/22
Placenta
T47D
MCF7
OVCAR3
OVSAHO
OVCAR8
COV362
OVCAR4
SNU119
GAPDH
GAPDH
